# Gamma Irradiation Effects on Fatty Acid Profiles, Adhesion, Plasmid Stability, and Secreted Proteins in *Shigella*: In Silico Insights into Post-Irradiation Virulence

**DOI:** 10.3390/microorganisms14091986

**Published:** 2026-09-08

**Authors:** Ali Ellafi, Najla Haddaji, Mabrouk Horchani, Karima Bekir, Rihab Lagha, Amina Bakhrouf, Eduardo Alberto López-Maldonado, Sonia Ben Younes

**Affiliations:** 1 Laboratory of Analysis, Treatment and Valorization of the Pollutants of the Environment and Products, Faculty of Pharmacy, University of Monastir, Street Avicenne, Monastir 5000, Tunisia; najla_haddaji@yahoo.fr (N.H.); karima_rokbani@yahoo.fr (K.B.); amina.bakhrouf@fphm.rnu.tn (A.B.); 2 Faculty of Sciences of Gafsa, University of Gafsa, University Campus Sidi Ahmed Zarroug, Gafsa 2112, Tunisia; 3 Laboratory of Heterocyclic Chemistry, Natural Products and Reactivity, Medicinal Chemistry and Natural Products (LR11ES39), Department of Chemistry, Faculty of Science of Monastir, University of Monastir, Avenue of Environment, Monastir 5019, Tunisia; 4 Research Unit UR17ES30: Virology and Antiviral Strategies, Higher Institute of Biotechnology, University of Monastir, Monastir 5000, Tunisia; rihablagha@yahoo.fr; 5 Faculty of Chemical Sciences and Engineering, Autonomous University of Baja California, Tijuana CP 22390, Baja California, Mexico; 6 Laboratory of Population Health, Environmental Aggressors and Alternative Therapies (LR24ES10), Faculty of Medicine of Tunis, University of Tunis-El Manar, Tunis 1068, Tunisia; benyounessonia@gmail.com

**Keywords:** *Shigella sonnei*, gamma irradiation, adherence, proteins secreted, plasmids, fatty acids

## Abstract

This study investigated the adaptive mechanisms of *Shigella sonnei* ATCC25931 in response to gamma irradiation stress (0.5 and 1 kGy), with a specific focus on the involvement of fatty acids (FAs) in membrane lipid composition remodeling, adherence, and extracellular proteins. The results demonstrated a notable enhancement in cell hydrophobicity and adherence to KB cells following irradiation, although the invasion rate exhibited a decline from 14% to 2.35% at 1 kGy. Furthermore, gamma irradiation induced notable alterations in fatty acid (FA) composition, characterized by a pronounced reduction in the unsaturated/saturated FA ratio. Additionally, the plasmid profiles revealed the loss of several original plasmids after gamma irradiation. The alterations in extracellular proteins were observed through SDS-PAGE, which demonstrated a reduction in expression at 1 kGy. Furthermore, a molecular docking analysis indicated that fatty acids play a role in inhibiting VirF, a key regulator of *Shigella* virulence. The least effective inhibitor was capric acid, while linoleic and gamma-linolenic acids formed the most stable inhibitory complexes, which prevented VirF from activating the virulence system. These alterations in fatty acids, plasmids, and extracellular proteins constitute adaptive responses to irradiation-induced stress.

## 1. Introduction

*Shigella*, a pathogen that affects humans and other primates, is the causative agent of bacillary dysentery, a significant global health issue, particularly in regions with poor hygiene, such as war zones and refugee camps [1,2,3]. Clinical symptoms typically appear 1 to 3 days after infection, often beginning with sudden, brief diarrhea. Shigellosis is an intestinal infection characterized by frequent bloody, mucus-laden stools, accompanied by abdominal cramps and rectal tenesmus. Fever is a constant symptom, often exceeding 39 °C. In severe cases, particularly among young children, diarrhea can lead to hospitalization due to toxicosis and/or severe dehydration, which may result in death.

Transmission occurs primarily through the fecal–oral route, either directly via human-to-human contact through contaminated hands or indirectly through polluted food, fomites, or water sources. While the disease typically has a sudden onset [4,5], gradual manifestations have also been reported [6]. Seasonal variations in shigellosis incidence are influenced by factors such as climate, hygiene practices, and living conditions. Increased mortality associated with *Shigella* infections is often linked to inadequate access to clean water, poor sanitation, malnutrition, and the high cost of antibiotic treatments. Numerous outbreaks globally underscore the critical role of waterborne transmission in the spread of *Shigella* [5,7].

Despite advances in water treatment and microbial control, water contamination remains a challenge, even in developed countries [8]. Wastewater treatment remains to be a global bottleneck, although various processes—mechanical, chemical, thermal, and biological—are available to mitigate these issues [9,10]. Among these, radiation technology has proven effective in pathogen inactivation, organic pollutant oxidation, and odor elimination. However, its widespread adoption is hindered by safety concerns and limited public awareness [11,12,13]. Gamma irradiation, in particular, has shown promise in sterilizing microorganisms and preventing decay [14].

Gamma irradiation damages cellular DNA through two primary mechanisms: (i) direct physical effects of ionizing radiation producing primary free radicals, and (ii) indirect biochemical effects involving reactive oxygen species that result in DNA double-strand breaks, single-strand breaks, or base pair substitutions. These mutations can lead to the conversion of pyrimidine bases to derivatives, such as 5-(hydroxymethyl)uracil, 5-formyluracil, 5-hydroxycytosine, and 5-hydroxyuracil [15].

Given the limited information available on the effects of gamma irradiation on *Shigella*, we conducted a laboratory experiment to assess its impact on *S. sonnei*. Our study aims to investigate key bacterial properties, including adhesive and invasive capabilities, plasmid dynamics, membrane fatty acid composition, and secreted protein profiles.

## 2. Materials and Methods

### 2.1. Bacterial Strain

This study utilized a particular strain of *Shigella*, *S. sonnei* ATCC 25931, which was sourced from Monastir Hospital in Tunisia and stored at −80 °C in Luria–Bertani (LB) broth supplemented with glycerol (15% *v*/*v*). To start the experimental cultures, the cells were allowed to grow in a tryptic soy broth (TSB, Difco, Saint-Ferréol, France) for 24 h at 37 °C.

### 2.2. Irradiation Treatment

*S. sonnei* bacterial strains were cultivated at 37 °C in a tryptic soy broth (TSB) under shaking conditions (150 rpm). Cultures of *Shigella* strains grown to the late logarithmic phase were divided into 1 mL aliquots without changing the broth. These aliquots were then exposed, in triplicate, to gamma irradiation using a cobalt-60 irradiator (point source, AECL, IR-79, Technopole, Sidi Thabet, Tunisia) on ice. The applied doses were 0.5 kGy and 1 kGy. Control cells were maintained on ice for the duration of the irradiation process. Following irradiation, both irradiated and non-irradiated *Shigella* cultures were diluted 50-fold using 50 mL of fresh medium and incubated at 37 °C with shaking (150 rpm) for 24 h before further analysis.

### 2.3. Cell Surface Hydrophobicity

Microbial hydrophobicity is a key virulence factor that facilitates the adhesion of microorganisms to host tissues [16]. Previous studies have demonstrated a significant correlation between surface hydrophobicity and the ability of microorganisms to adhere to epithelial cells in vitro [17]. Therefore, it was crucial to investigate the hydrophobicity of both irradiated and non-irradiated *Shigella* strains in this study. The hexadecane partitioning method was employed to evaluate hydrophobicity [16]. Bacterial cells were grown in a tryptic soy broth overnight, washed, and suspended in 4 mL of phosphate-buffered saline (PBS). The initial absorbance of the cells was measured (OD_540_). After adding 1 mL of hexadecane to each cell suspension, the mixture was allowed to equilibrate for 10 min. The suspensions were then incubated again at 37 °C for 30 min. The water layer was removed and aerated to eliminate hexadecane traces. The final absorbance (OD_540_) was measured against a hexadecane-extracted PBS blank. The hydrophobicity index was calculated by dividing the hexadecane-extracted sample’s absorbance by the sample’s absorbance before extraction.

### 2.4. Culture Cell Adherence and Invasion Assays

This research aimed to evaluate the attachment of *Shigella* strains to the human oral cavity epidermoid carcinoma KB cell line. The KB cells were cultured in a 96-well microtiter plate containing a minimal essential medium (MEM) supplemented with Earle’s salts and 10% fetal bovine serum. The culture was incubated overnight at 37 °C in a 5% CO_2_ atmosphere. Simultaneously, *Shigella* strains were grown in TSB using a rotary shaker at 37 °C and 150 rpm. The protocol followed was adapted from Ellafi et al. (2009) [18]. Bacterial cells were washed three times with a serum-free MEM and centrifuged at 6000× *g* for 15 min, then resuspended in the same medium. The bacterial suspension was adjusted to a concentration of 10^8^ CFU/mL.

For the adherence assays, KB cells were exposed to *Shigella* strains at a concentration of 10^8^ CFU/mL and incubated at 37 °C with 5% CO_2_ for one hour. After incubation, the bacterial suspension was carefully removed to eliminate unattached bacteria. The KB cell monolayers were washed three times with Dulbecco’s Modified Eagle Medium (DMEM). Subsequently, 1 mL of Triton X-100 in PBS was added to the wells and left for five minutes at room temperature to release attached bacteria from the cells.

To investigate bacterial invasion, *Shigella* strains were allowed to invade the KB cells during a two-hour incubation at 37 °C in a 5% CO_2_ environment. Following this, extracellular bacteria were removed by aspirating the supernatant, and the cells were washed with DMEM. Intracellular bacteria were quantified by plating serial dilutions after lysing the KB cells with a 1% Triton X-100 PBS solution. All experiments were performed in triplicate [18].

### 2.5. Plasmid Extraction and Analysis

Plasmid DNA extraction was performed using the BB Plasmid MiniPREP Kit (Bio Basic; Product Code: BMPK00100; Singapore), following the manufacturer’s instructions. This kit offers a rapid, simple, and cost-effective method for isolating plasmid DNA from cultured bacterial cells. The procedure is based on the alkaline lysis of bacterial cells, followed by DNA binding to a glass fiber matrix in the spin column under high-salt conditions. This protocol eliminates the need for phenol extraction or ethanol precipitation. The purified plasmid DNA was eluted in a small volume of Tris buffer (provided in the kit) or water with a pH between 7.0 and 8.5. The isolated DNA is of a high quality and suitable for various downstream applications, including restriction enzyme digestion, sequencing, library screening, in vitro translation, robust cell transfection, ligation, and transformation. The entire process was completed in approximately 20 min. To confirm the presence of plasmids, electrophoresis was performed on a 0.7% agarose gel containing 0.5 μg/mL ethidium bromide. The gel was visualized under ultraviolet light, and images were captured for documentation.

### 2.6. Analysis of Fatty Acids

The fatty acids present in the cells were analyzed via MIDI protocols on cells taken from 10 mL of each cell suspension [18,19]. To minimize hands-on time, all saponification, methylation, extraction, and washing reagents were dispensed with autopilots into the same tube. After extraction, the samples were analyzed via gas chromatography via a Hewlett Packard HP 5890 capillary gas chromatograph connected to an HP ChemStation integrator. To identify the fatty acid methyl esters, external standards that underwent the same process as the biological samples were used. The results are presented as the area percentage of total fatty acids. To differentiate *Shigella* cell membrane fatty acids under different conditions, we used total saturated fatty acids (SFAs) and unsaturated fatty acids (UFAs). The UFA/SFA ratio indicates the degree of membrane fluidity, where higher ratios represent greater fluidity [18].

### 2.7. Extracellular Protein Extraction

The extracellular proteins of *Shigella* were prepared by cultivating cells in TSB prepared with seawater until they reached an optical density of 0.6 at 600 nm. Bacterial cells were eliminated from cultures through centrifugation and filtration, and proteins from the cell-free culture supernatants were precipitated and recovered [4]. The pellets were resuspended, precipitated, washed, dried, and resuspended in PBS. Two micrograms of secreted proteins were analyzed via SDS-PAGE with a 15% acrylamide separating gel and a 5% acrylamide stacking gel. The proteins were visualized via Coomassie brilliant blue from Sigma, and the molecular weights were determined via commercial markers (High-Range Rainbow) from Amersham [4].

### 2.8. Molecular Identification of Stressed Bacteria

To assess the impact of gamma irradiation on *S. sonnei* strains, a polymerase chain reaction (PCR) assay was employed to detect the presence of the *ipaH* gene. This gene, which encodes the invasion plasmid antigen H (*ipaH*), is a key marker for the diagnosis of dysentery and is carried by all four *Shigella* species [19]. Bacterial strains were initially cultured on TSA at 37 °C for 24 h. A single colony was transferred to TSB and incubated at 37 °C for an additional 24 h. A 1.5 mL aliquot of the culture was centrifuged, and the DNA was extracted by boiling for 5 min, followed by centrifugation at 13,000 rpm for 8 min. The supernatant served as the template for PCR amplification using primers specific to the *ipaH* gene. The PCR reaction was performed in a 25 µL volume containing the following components: 50 ng of extracted DNA, 5 µL of 5× Green GoTaq buffer, 0.25 µL of dNTPs (10 mM), 0.5 µL of MgCl_2_ (50 mM), 1 µL each of *ipaH* forward primer (5′-GTTCCTTGACCGCCTTTCCGATAC-3′; 25 pM) and reverse primer (5′-CATTTCC TTCACGGCAGTGGA-3′; 25 pM [20], and 1 U of GoTaq DNA polymerase (Promega, Charbonnières, France). Amplification was conducted using a Bio-Rad Thermocycler PTC 100 (Bio-Rad, Wilmington, Delaware, United States) under the following conditions: initial denaturation at 94 °C for 5 min, followed by 35 cycles of denaturation at 94 °C for 90 s, annealing at 57 °C for 30 s, and elongation at 72 °C for 90 s, with a final extension step at 72 °C for 10 min. PCR products (5 µL) were separated on a 1% agarose gel stained with ethidium bromide (0.5 µg/µL). Electrophoresis was conducted at 90 V for 1 h, and bands were visualized under ultraviolet transillumination. The amplified products sizes were compared against a 100-bp molecular size marker (Promega, Madison, WI, USA) and photographed for documentation.

### 2.9. Molecular Docking Procedure

Molecular docking was performed via the AutoDock 4.2 program package [21]. The crystal structure of “ETEC Rns bound to a potential inhibitor, decanoic acid” (PDB: 6XIU) [22] was obtained from the RSCB protein data bank (https://www.rcsb.org/, accessed on 23 September 2024). Prior to initiating the docking process, all water molecules were removed from the structure, and missing hydrogen atoms were added along with Gasteiger charges during the preparation of the receptor input file. The AutoDock tools were used to prepare all compound and protein files in PDBQT format. Grid maps were precalculated using AutoGrid to reduce computational time during docking. The geometric optimization of all compounds was performed using ACD/3D Viewer software (ACD/Labs 2017.2.1 (File Version D40E41, Build 93524, 21 Apr 2017) (http://www.filefacts.com/acd3d-viewer-freeware-info, accessed on 23 September 2024). Molecular interaction visualization between the ligands and the receptor was achieved with Discovery Studio 2017R2 (https://www.3ds.com/products/biovia/discovery-studio/visualization, accessed on 23 September 2024).

### 2.10. Statistical Analysis

Data analysis was conducted on a Windows OS using the statistical software SPSS 13.0. The degree of adhesion was compared via the Friedman test, and the significance of the results was evaluated via the Wilcoxon signed-rank test. Significant differences were identified when *p* values were less than 0.05.

## 3. Results

### 3.1. Effect of Stress on the Surface Hydrophobicity

Gamma irradiation significantly impacted the surface hydrophobicity of *S. sonnei* (Table 1). All *Shigella* strains presented a significant increase (*p* < 0.05) in surface hydrophobicity when subjected to starvation. There was a 15% to 35% increase in hydrophobicity after this stress at 1kGy.

### 3.2. Adherence and Invasion Assays of KB Cells

Adherence assays to cultured cells were conducted using a KB cell line. Initially, we observed that untreated *S. sonnei* (0.7%) exhibited weak adhesion to KB cells (Table 1). Following gamma irradiation, a significant increase (*p* < 0.05) in the adhesion percentage was noted at 0.5 kGy. However, in the invasion assays, a significant decrease (*p* < 0.05) in this percentage was observed after gamma irradiation at 1 kGy (2.35%) (Table 1).

### 3.3. Plasmid Analysis

**Figure 1:**
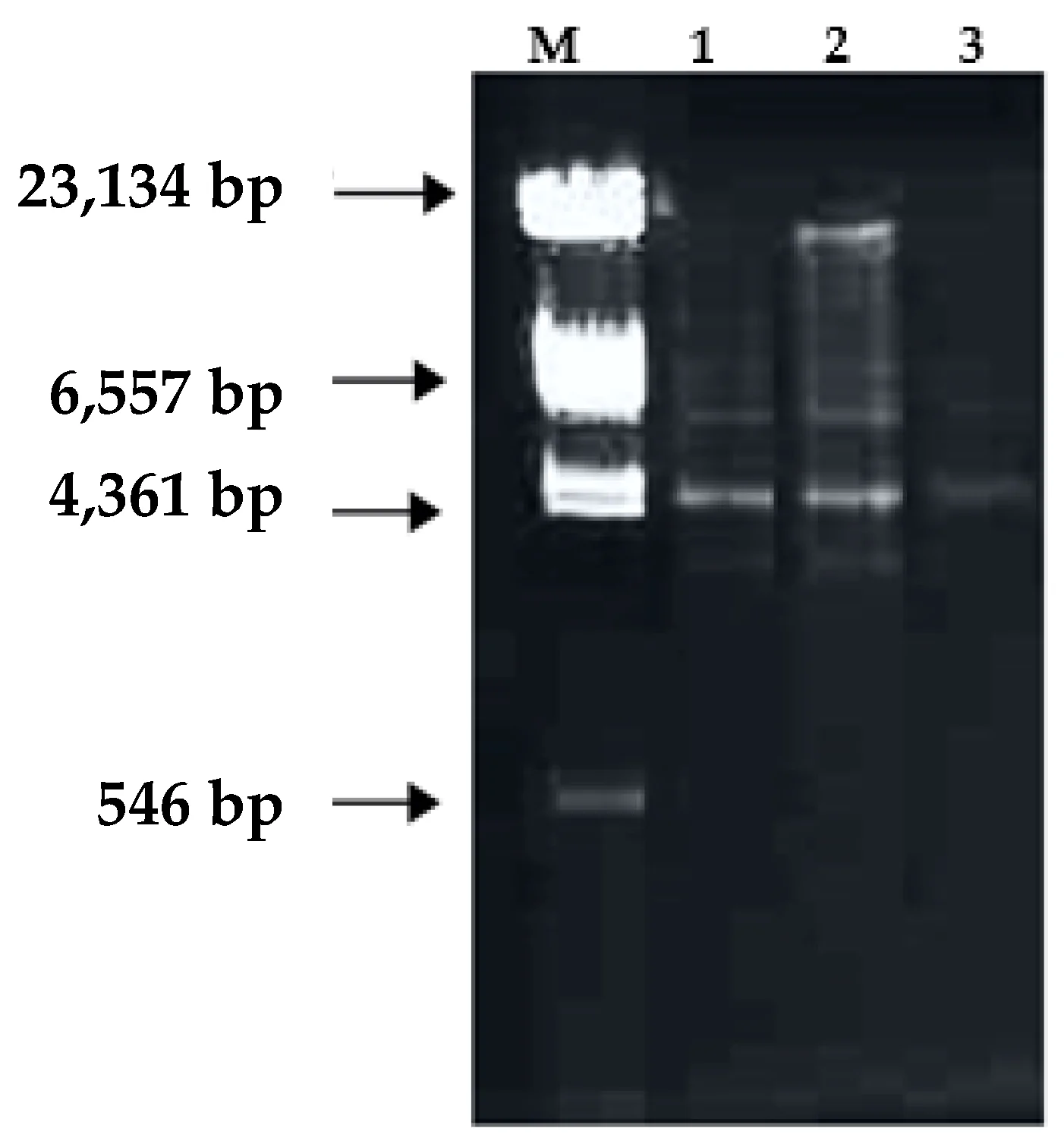
Plasmid DNA profiles of *S. sonnei* exposed to gamma irradiation. M, molecular weight marker; Lane 1: strain irradiated at 0.5 kGy; Lane 2: strain non-irradiated; Lane 3: strain irradiated at 1 kGy.

*S. sonnei* initially possesses four plasmids, ranging in size from 3 to 23 kb. However, after irradiation, many of the original plasmids of the strains were lost. Specifically, 9 kb plasmids were lost in cells irradiated at 0.5 kGy, and 9 kb, 6 kb, and 3 kb plasmids were lost in cells irradiated at 1 kGy (Figure 1).

Mobile genetic elements, such as bacteriophages, insertion sequences, and plasmids, play a crucial role in the evolution and genomic plasticity of bacterial pathogens, including *Shigella sonnei*. In *S. sonnei*, plasmids encode essential virulence factors regulated by genes located on the virulence plasmid (VP) as well as the chromosome [23].

*S. sonnei* initially harbors four plasmids, ranging in size from 3 to 23 kb. However, gamma irradiation led to the loss of several original plasmids. Specifically, 9 kb plasmids were lost in cells irradiated at 0.5 kGy, while plasmids of 9 kb, 6 kb, and 3 kb were lost in cells exposed to 1 kGy (Figure 1). This loss of plasmids after irradiation highlights the potential impact of gamma irradiation on the genetic structure and virulence potential of *S. sonnei*.

### 3.4. Membrane Fatty Acid Composition of S. sonnei

In this study, the fatty acid composition of *S. sonnei* cells was examined under different growth conditions via chromatographic methods. The analysis revealed twenty fatty acids in cells grown under normal and stress conditions (Table 2). The five prominent peaks identified were palmitic acid (C16:0), palmitoleic acid (C16:1n7), n-hexadecenoic acid (C16:1n5), vaccenic acid (C18:1n7), and linoleic acid (C18:2n6), whereas fifteen other fatty saturated and monounsaturated acids were detected at lower concentrations. When *Shigella* cells were exposed to gamma irradiation, significant changes in membrane fatty acid composition were observed (Table 3). There was a decrease in saturated fatty acids such as C15 iso, C16, C17, C17 iso. and C18, as well as mono- and polyunsaturated fatty acids including C14:1, C17:1, C18:1n9, C18:2n6, and C18:3n6 (Table 2).

Conversely, there was an increase in saturated fatty acids, such as C15 anteiso and C17 anteiso, and monounsaturated fatty acids, such as C16:1n7, C16:1n5, C18:1n7, and C20:1n9. Other fatty acids remained stable, with no significant changes in content. The UFA/SFA ratio was higher in the 1 kGy irradiated strain and lower in the control strains (Table 3).

### 3.5. Extracellular Protein Analysis

**Figure 2:**
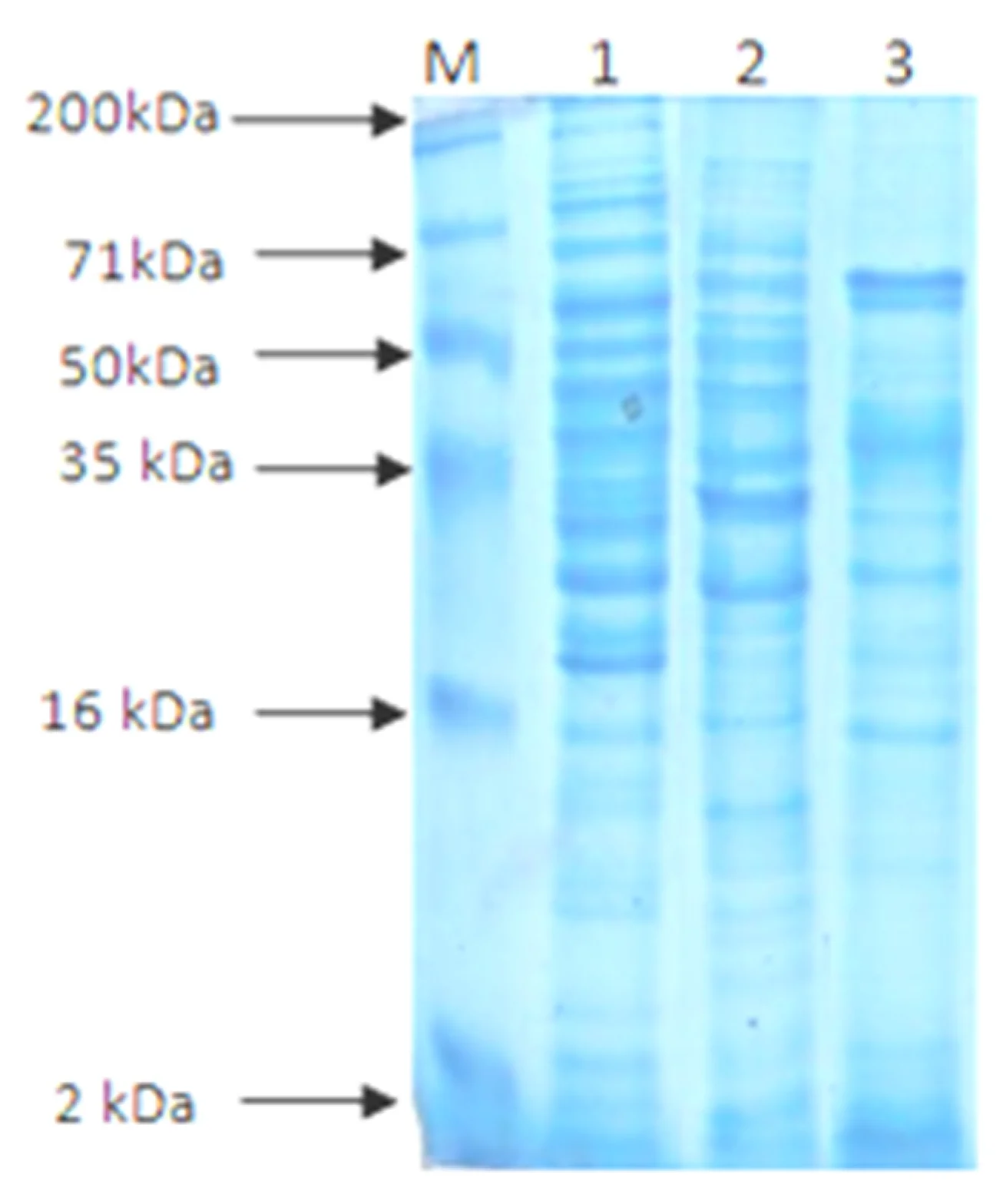
Extracellular proteins of *S. sonnei* exposed to irradiation. M, molecular weight marker; Lane 1: non-irradiated strain; Lane 2: strain irradiated at 0.5 kGy; Lane 3: strain irradiated at 1 kGy.

The extracellular proteins of *S. sonnei* were analyzed before and after gamma irradiation via SDS-PAGE (Figure 2). *Shigella* is known to secrete a substantial number of proteins into the extracellular medium. After gamma irradiation, a significant difference was observed, particularly after irradiation at 1 kGy. There was a noticeable decrease in the expression of almost all proteins compared with those in the normal state and in the cells irradiated at 0.5 kGy. Consequently, two bands corresponding to molecular weights of approximately 60 and 58 kDa presented altered expression levels after gamma irradiation at 1 kGy.

### 3.6. Molecular Confirmation of the Stressed Strains

**Figure 3:**
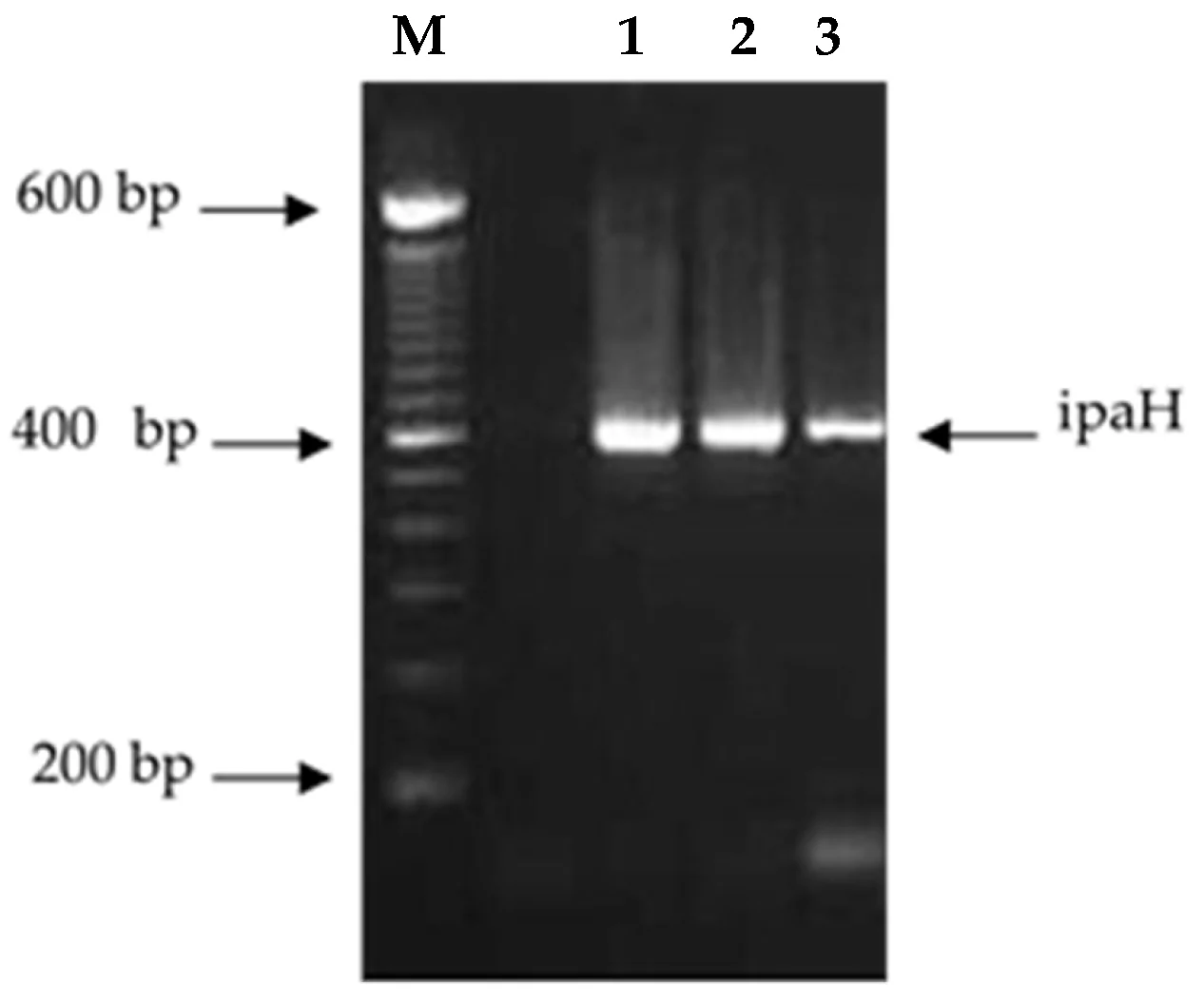
Process of agarose gel electrophoresis involving amplification of the ipaH gene via polymerase chain reaction (PCR). The DNA molecular size marker M represents a sample size of 100 bp. The three subsequent lanes on the gel represent the PCR amplicons from the DNA amplification of *S. sonnei*. Lane 1 represents a non-irradiated strain; Lane 2 represents a strain irradiated at 0.5 kGy; Lane 3 represents a strain irradiated at 1 kGy.

PCR was employed to confirm the identity of the gamma irradiated *S. sonnei* strains by amplifying the ipaH gene (400bp) (Figure 3). The expression of the ipaH gene is significantly reduced in the 1 kGy irradiated *S. sonnei* strain compared to both the control strain and the 0.5 kGy irradiated strain. This clearly shows that irradiation has affected the expression of the ipaH gene.

### 3.7. Molecular Docking Analysis

**Figure 4:**
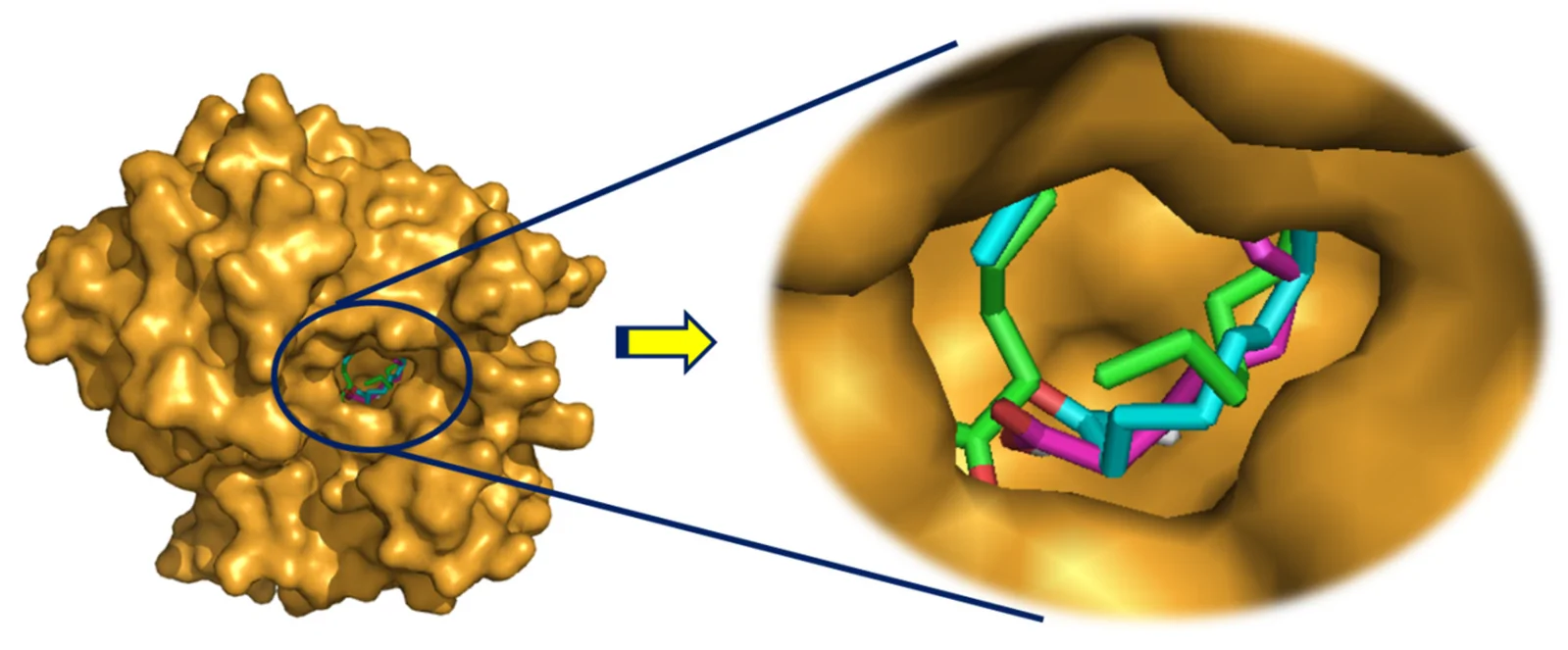
3D binding modes of the docked ligands: capric acid (purple color), linoleic acid (cyan color), and gamma-linolenic acid (green color) in the binding cavity of ‘VirF’ (the red color indicates the presence of an oxygen atom in each molecule).

The investigation focused on whether *Shigella* virulence could also be affected through VirF-compound interactions in the intestinal environment. To test this hypothesis, we carried out an accurate structural prediction of the VirF receptor (pdb: 6xiu) and then chose, via molecular docking simulations, a group of fatty acids as candidate ligands. Figure 4 shows that the selected docked ligands fit well in the binding cavity of the target protein.

**Figure 5:**
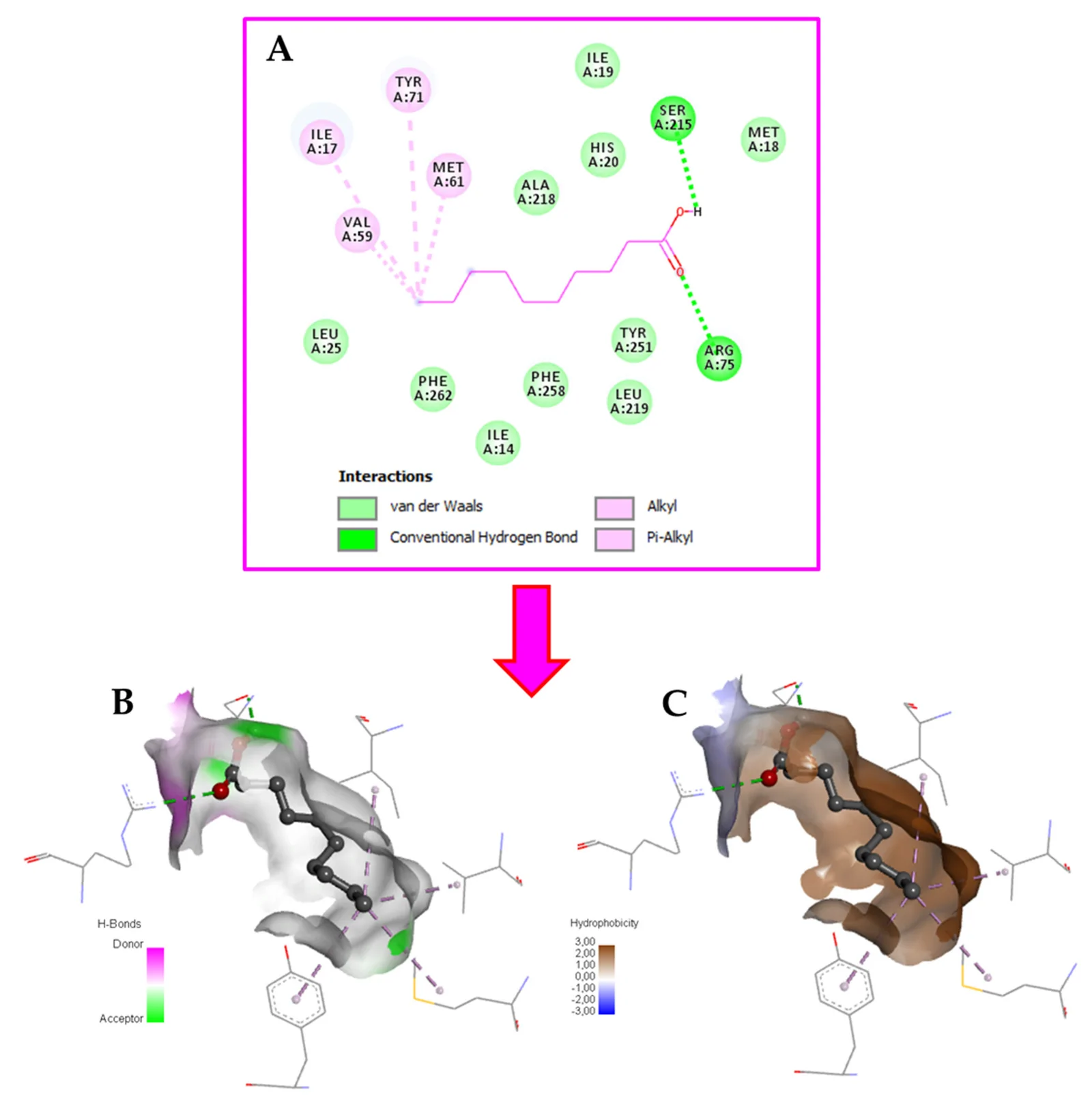
2D binding modes of the docked compounds: (**A**) capric acid; (**B**) H−bond cavity of capric acid; (**C**) hydrophobic cavity of capric acid within the binding site of ‘VirF’.

According to the results shown in Table 4, among all the tested fatty acids, capric acid (also considered the co-crystallized ligand with VirF (pdb: 6xiu)) with the highest energy (−5.0 kcal/mol) was found to be the least effective ligand as a VirF inhibitor. This ligand is involved in two conventional hydrogen bonds with Arg75 and Ser215 in addition to four hydrophobic interactions: three Alkyl with Ile17, Val59, Met61 and a Pi-Alkyl with Tyr71 in addition to some van der Waals interactions (Figure 5). Figure 5B of the H-bond cavity shows that the green area (acceptor) is around the acid function (COOH), and the hydrophobic cavity (Figure 5C) displays a brown color around the alkyl chain of the molecule; however, in this case, the ligand (capric acid) is not surrounded by this cavity.

**Figure 6:**
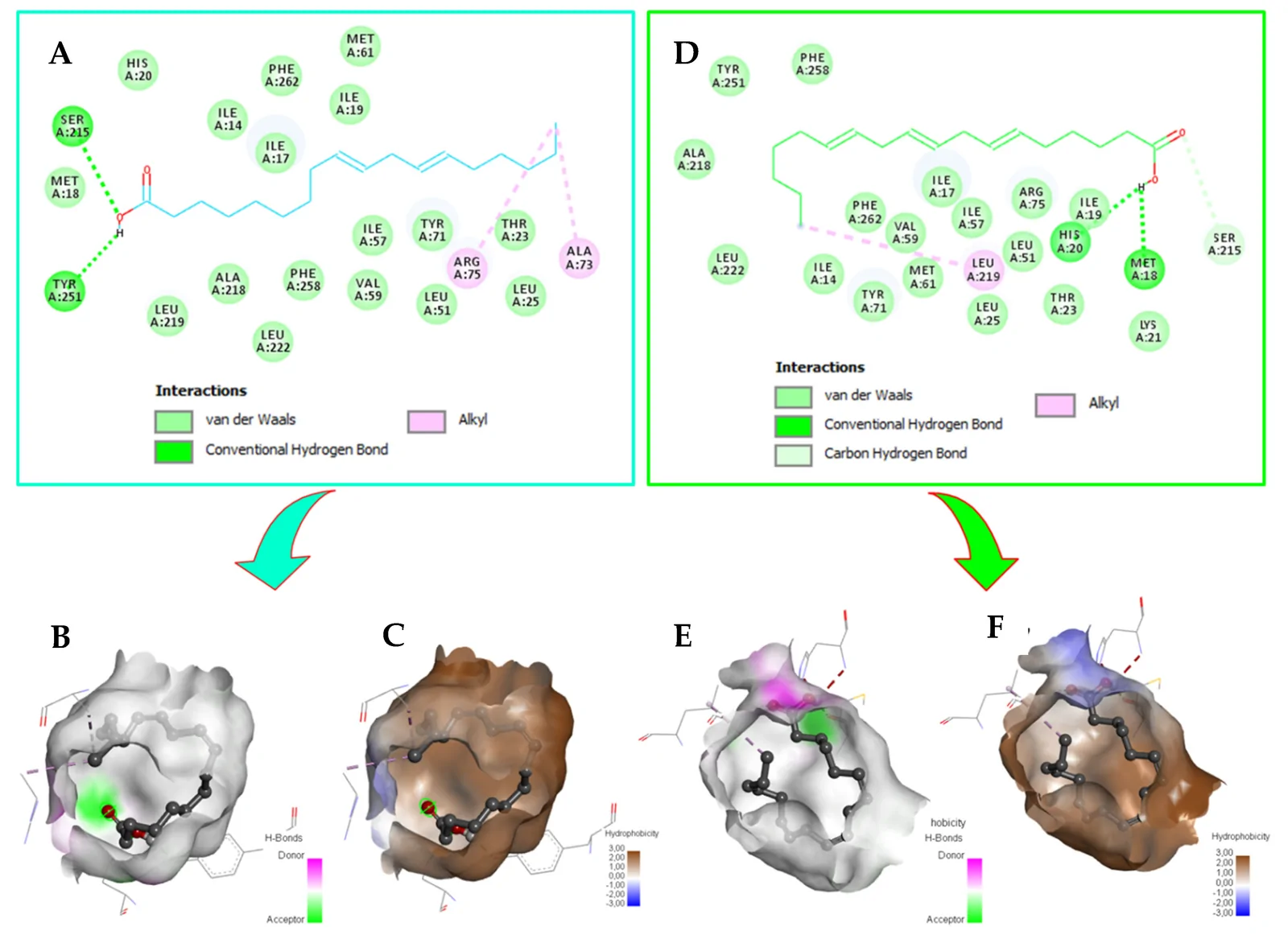
2D binding modes of the docked compounds: (**A**) linoleic acid; (**B**) H−bond cavity of linoleic acid; (**C**) hydrophobic cavity of linoleic acid; (**D**) gamma−linolenic acid; (**E**) H−bond cavity of gamma−linolenic acid; (**F**) hydrophobic cavity of gamma−linolenic acid within the binding site of ‘VirF’.

As shown in Figure 6C,F, where both molecules, linoleic acid and gamma-linolenic acid, have more wrapped hydrophobic cavities with intense brown colors, the importance and interesting role of their long alkyl chains, which contain unsaturation (double bonds: C=C) in the inhibitory potential of VirF and therefore their power against *Shigella* are shown. These two ligands, that display the best docking scores (−6.5 kcal/mol), formed a stable complex with the target receptor. This stability is perceptible through the formation of numerous van der Waals interactions in addition to other contacts. Specifically, this docked ligand is involved in two H-bonds with Ser215 and Tyr251, in addition to two Alkyl interactions with Ala73 and Arg75, whereas its analog gamma-linolenic acid forms a carbon hydrogen bond with Ser215, Alkyl with Leu219, and two H-bonds with Met18 and His20 (Figure 6A,D).

## 4. Discussion

The survival of bacteria depends on their ability to sense and respond to environmental changes. Our study revealed that, after exposure to gamma irradiation, the cell surface hydrophobicity and adhesion of *S. sonnei* strains to line cells or polystyrene increased significantly. The ability to stick to surfaces is one of the factors that contributes to the harmfulness of microorganisms to host tissue. Research has indicated that there is a correlation between surface hydrophobicity and in vitro adherence to epithelial cells. Hence, bacteria tend to stick to surfaces as a survival mechanism. The bacterial colonization of solid surfaces is a fundamental bacterial strategy in various environments [16,19]. Invasion assays revealed a notable decrease in the percentage of *S. sonnei* cells invading KB cells following gamma irradiation at 1 kGy. The environmental changes induced by irradiation significantly alter bacterial function and protein expression. As a consequence of these adverse effects, the synthetic functions of the cells are inhibited, and cell division is interrupted [19]. Furthermore, gamma irradiation inflicts damage to the DNA of cells [23,24,25]. Our results also demonstrated that the strains experienced a loss of many original plasmids after irradiation, with a particular emphasis on the loss of the large plasmid, which contributed to the observed decrease in invasiveness. Consequently, the reduction in the invasion of line cells can be directly attributed to a significant and relevant decline in the expression of numerous genes and membrane alterations. These findings align with a study on *Vibrio* cells, where gamma irradiation induced morphological alterations in the membranes of stressed cells [19].

Our research suggests that gamma irradiation is the leading cause of changes in the composition of membrane fatty acids in *S. sonnei* and that it appears to affect membrane fluidity. Furthermore, this irradiation leads to membrane adaptation, as evidenced by the decrease in the UFA/SFA ratio. Prior studies have shown that a reduced UFA/SFA ratio is linked to decreased membrane fluidity, which is also observable in *Shigella* cells [26,27]. It is generally admitted that cells regulate their lipid composition to achieve a degree of fluidity compatible with life [26].

On the other hand, our results do not show a significant variation between the profiles of the non-irradiated strain, and the strain irradiated at 0.5 kGy. The profile of the strain irradiated at 1 kGy shows reduced expression of almost all the proteins from the normal state and at 0.5 kGy. These changes at the level of secreted protein profiles confirm those found by studying invasion. Indeed, blocking gamma radiation, even transiently, affects essential replication and transcription processes by damaging DNA, proteins, and lipids [16]. According to Ellafi et al. [18], radiation can cause irreversible changes in protein conformation, such as fragmentation or aggregation. Similarly, Caillet et al. [27] reported that gamma radiation affects the synthesis of Hsps in bacterial foodborne pathogens in a manner dependent on the radiation dose.

Various bacteria can be dangerous to human health, and they have virulence mechanisms that allow them to cause infections. These mechanisms include the secretion of toxins, such as enterotoxin, hemolysin, and cytotoxin, as well as enzymes, such as protease and lipase. Researchers have reported that, when three specific genes (vvhA, ctxA, and tdh) are expressed in three different types of *Vibrio* bacteria (*V. vulnificus*, *V. cholera*, and *V. parahaemolyticus*), and when they are exposed to gamma irradiation at a dose of 0.5 or 1 kGy, the expression of these genes decreases significantly. The reduction in mRNA levels ranged from 0.3- to 0.001-fold, which translates to a 3- to 1000-fold decrease in gene expression. As gamma irradiation can reduce the virulence of pathogens, it can also increase their virulence [28]. DNA molecules can be heavily damaged by radiation treatment, which prevents them from functioning normally. Ionizing radiation is a physical agent that directly targets DNA molecules by producing free radicals and reactive oxygen species or through direct interactions. Bacterial cells respond to radiation in various ways, such as by halting cell cycle progression and repairing DNA lesions [27].

On the basis of our research, it appears that gamma irradiation is the predominant contributor to the changes observed in the membrane fatty acid composition of *S. sonnei*, resulting in a shift in membrane fluidity [26]. This alteration in irradiated cells is manifested by a decrease in the ratio of unsaturated fatty acids to saturated fatty acids (UFA/SFA) [19,29]. Previous studies have emphasized the importance of regulating the lipid composition to attain the necessary membrane fluidity for ensuring survival [27].

Alterations in membrane fatty acid composition in microorganisms have far-reaching consequences, notably in modulating the activity of intrinsic proteins responsible for crucial functions, such as ion pumping and nutrient uptake [29]. In our study, the profile analysis revealed no significant variation between the non-irradiated strain, and the strain exposed to 0.5 kGy. However, the expression of almost all the proteins in the strain irradiated at 1 kGy was lower than that in the normal and 0.5 kGy conditions. These changes in secreted protein profiles align with our invasion study findings, underscoring the impact of gamma radiation on essential cellular processes, such as replication and transcription, causing damage to DNA, proteins, and lipids [25].

Our results also demonstrated that the strains experienced a significant loss of original plasmids after irradiation, with particular emphasis on the large plasmid. This loss contributed to the observed decrease in invasiveness. The reduction in the invasion of cell lines can be directly attributed to a notable decline in the expression of various genes and alterations in membrane composition. Indeed, in many pathogenic bacteria, large plasmids play a critical role in virulence and the dissemination of antimicrobial resistance [30].

While plasmids confer beneficial traits under certain conditions, they often impose a substantial metabolic cost on the host cell. The genetic plasticity inherent to many large plasmids enables rearrangements that facilitate the appropriate expression of genes, thereby allowing bacterial adaptation to new ecological niches [31]. For instance, the *S. sonnei* virulence plasmid (pINV) serves as a prime example of how genetic events drive plasmid evolution, reflecting bacterial adaptation to new environments and lifestyles [24,32,33].

The work of Ellafi et al. [18] has emphasized the potential for radiation to induce irreversible changes in protein conformation, leading to phenomena such as fragmentation or aggregation. Similarly, Caillet et al. [27] have demonstrated that gamma radiation affects the synthesis of heat shock proteins (Hsps) in foodborne bacteria, with the response being dependent on the radiation dose. Many bacteria that are pathogenic to humans deploy virulence mechanisms involving the presence of enterotoxins, hemolysins, cytotoxins, and various enzymes, such as proteases and lipases. Notably, the transcriptional changes observed in key virulence-related genes (vvhA (*V. vulnificus*), ctxA (*V. cholera*), and tdh (*V. parahaemolyticus*)) of *Vibrio* species after exposure to gamma irradiation resulted in a substantial reduction in mRNA levels, ranging from 0.3- to 0.001-fold under optimal expression conditions for 15–18 h. This corresponds to a 3- to 1000-fold reduction in mRNA levels, suggesting the potential of gamma irradiation both to reduce and to increase the virulence of pathogens, depending on specific conditions [28].

Radiation treatment can harm DNA molecules, leading to their faulty function. Ionizing radiation, as a physical agent, can directly interact with DNA molecules or produce free radicals and reactive oxygen species. Bacterial cells exhibit various reactions when exposed to radiation, such as pausing the cell cycle and initiating DNA lesion repair [27].

Fatty acids (FAs) are essential in regulating a variety of biological functions across both prokaryotic and eukaryotic systems, from maintaining membrane stability to facilitating key cellular activities, like differentiation, proliferation, secretion, and invasion. In bacterial cells, FAs can disrupt cellular processes, leading to cell death or growth inhibition by targeting multiple cellular components [34]. Recent studies, particularly molecular docking analyses, have shed light on the influence of FAs on bacterial virulence factors, specifically focusing on the *Shigella* VirF protein [35].

VirF, a master regulator of the virulence system in *Shigella*, plays a pivotal role in controlling the expression of genes that enable the pathogen to invade and survive within host cells. Without its activation, *Shigella* loses its capacity to initiate infection, making VirF an attractive target for strategies aimed at reducing the pathogen’s virulence and limiting its impact on human health [34,35,36,37].

Belonging to the AraC/XylS family of proteins, VirF shares functional similarities with other virulence regulators, such as *Vibrio cholerae* ToxT, enterotoxigenic *E. coli* (ETEC) Rns, and *Salmonella enterica* RtsA, HilC, and HilD [38]. These proteins are integral to virulence expression in related pathogens and are known to be inhibited by specific compounds, including fatty acids [39]. 

Given its essential role and tight regulation, VirF has been extensively studied, leading to the identification of numerous mechanisms governing its activity [40]. Targeting VirF not only holds promise for mitigating *Shigella* infections but provides insights into broader strategies for managing virulence in related bacterial pathogens.

The molecular docking studies have explored how different fatty acids interact with the VirF protein. The results showed that when *Shigella* is in an environment with low free fatty acids, VirF tends to bind and release linoleic acid and oleic acid more easily. This allows the virulence system to be activated, promoting the invasive potential of *Shigella*. However, when exposed to saturated fatty acids like myristic, stearic, and palmitoleic acids, VirF activity is more effectively inhibited, preventing the virulence genes from being expressed [35].

In this study, capric acid, a medium-chain fatty acid, was found to be the least effective in inhibiting VirF, resulting in weaker suppression of the virulence system. On the other hand, unsaturated fatty acids, such as linoleic acid and gamma-linolenic acid, were identified as the most effective inhibitors. They were shown to form stable complexes with VirF, altering its structure and function. This binding induces a structural transition in the VirF protein, causing it to shift from an “open” conformation, where it can activate virulence genes, to a “closed” form that renders it inactive [35,41]. Specifically, this conformational change prevents VirF from interacting with the VirB promoter, a region that initiates the expression of virulence genes in *Shigella*. This process could play a crucial role in the programmed control of *Shigella* virulence expression during its passage through the human intestine, where long- and medium-chain fatty acids are less available [35,41]. Notably, palmitoleic acid (PAO), which is present in human bile, acts as a potent inhibitor of virulence, suggesting that *Shigella* may repress its virulent state in the small intestine before reactivating it in the colon [35].

This structural transition is significant because it highlights a potential mechanism by which FAs regulate *Shigella* virulence as the bacterium passes through different environments in the human body. For instance, in the small intestine, where the concentration of long- and medium-chain fatty acids, like palmitoleic acid, is higher (due to bile and diet), VirF may be inhibited. This suggests that *Shigella* might repress its virulent state while passing through the small intestine, only to reactivate it later in the colon, where fatty acid concentrations are lower [42].

This FA-mediated regulation of VirF is not unique to *Shigella*. Similar mechanisms have been observed in other bacterial pathogens. For example, in *Vibrio cholerae*, FAs inhibit the ToxT protein, which controls the expression of the cholera toxin, and in *Escherichia coli*, FAs similarly affect Rns, a virulence regulator. The similarities in these regulatory pathways suggest that targeting these virulence proteins with FA-like inhibitors could be a broad-spectrum strategy against multiple pathogens [35].

The findings of this study open new possibilities for developing anti-virulence therapies. By focusing on inhibiting virulence rather than bacterial growth, such therapies could reduce the selective pressure for antibiotic resistance. Fatty acids or FA-mimicking molecules could be designed to specifically bind to VirF and other virulence regulators, offering a novel approach to treating bacterial infections like shigellosis. This strategy represents an alternative or complement to traditional antibiotics, particularly in the face of increasing antibiotic resistance in *Shigella* and other pathogens. The discovery of FA-mediated VirF inhibition underscores the potential for designing targeted anti-virulence drugs, marking a promising direction for future research and drug development [35].

## 5. Conclusions

This study examines the adaptive mechanisms employed by *Shigella sonnei* in response to gamma irradiation stress, focusing on the alteration in fatty acid composition, plasmid stability, adhesion properties, extracellular protein profiles, and molecular docking insights into virulence regulation. Gamma irradiation significantly remodeled membrane fatty acids, decreasing the unsaturated/saturated fatty acid ratio, which increased hydrophobicity and adherence while reducing invasion capacity at higher doses (1 kGy).

A plasmid analysis revealed the loss of specific plasmids post-irradiation, indicating genetic instability and potential attenuation of virulence. Irradiation also reduced the expression of extracellular proteins and the virulence-related ipaH gene. Molecular docking simulations showed that linoleic and gamma-linolenic acids effectively inhibit VirF, a master regulator of *Shigella* virulence, suggesting a novel approach for targeted antimicrobial strategies.

Overall, gamma irradiation triggers multiple adaptive responses in *S. sonnei*, including structural and functional changes that influence its virulence potential. The inhibitory effect of specific fatty acids on VirF highlights a promising strategy for targeting *Shigella* virulence.

These findings pave the way for further research. Exploring the broader effects of gamma irradiation on *Shigella*’s genetic material and virulence pathways could lead to innovative bacterial infection management strategies, especially where irradiation is used for disinfection. Investigating fatty acids in regulating bacterial virulence could inspire novel antimicrobial agents or adjunct therapies. Additionally, understanding long-term irradiation effects on bacterial populations, including resistance development, is critical for future infection control measures.

Future research should examine the broader implications of irradiation on related pathogens, optimize irradiation doses for maximum effectiveness, and integrate this method with current disinfection strategies. Fatty acid-based therapeutics could complement traditional antimicrobial treatments. These findings establish gamma irradiation as a non-chemical, effective method for mitigating *Shigella* virulence, with applications in infection control, water treatment, and food safety. Further investigation of irradiation parameters and fatty acid-based therapies is essential for advancing antimicrobial strategies.

## Figures and Tables

**Table 1:** Effect of gamma irradiation on surface hydrophobicity and on the capacity of *S. sonnei* to adhere to and invade KB cells.

	Strain Non-Irradiated	Strain Irradiated at 0.5 kGy	Strain Irradiated at 1 kGy
HI	15.000 ± 0.630	19.000 * ± 1.230	35.000 * ± 1.240
Adhesion%	0.700 ± 0.002	1.400 * ± 0.018	0.850 ± 0.004
Invasion%	14.000 ± 0.049	15.00 ± 0.016	2.350 * ± 0.014

*: *p* < 0.05.

**Table 2:** Fatty acid composition of *S. sonnei* exposed to gamma irradiation.

Fatty Acid (%)		Strain Non-Irradiated	Strain Irradiated at 0.5 kGy	Strain Irradiated at 1 kGy
SFA	C10	1.73	1.1	0.91
	C12	0.26	0.28	0.29
	C14	0.46	0.37	0.47
	C15 iso	0.44	0.05	0.06
	C15 anteiso	0.55	0.54	0.6
	C16	36.08	31.86	31.31
	C17	0.31	0.29	0.27
	C17 iso	1.16	0.9	0.79
	C17 anteiso	0.43	0.42	0.52
	C18	1.14	1.03	0.99
	C20	0.52	0.54	0.51
MUFA	C14:1	0.23	0.21	0.06
	C16:1w7	4.03	4.75	5.49
	C16:1w5	6.52	7.21	7.38
	C17:1	0.91	0.8	0.76
	C18:1w9	1.11	0.97	0.88
	C18:1w7	24.44	29.27	30.82
	C20:1w9	0.2	0.21	0.33
PUFA	C18:2w6	20.34	14.75	17.1
	C18:3w6	2.31	2.04	2.56

SFA: saturated fatty acid; MUFA: monounsaturated fatty acid; PUFA: polyunsaturated fatty acid. C10: capric acid; C12: lauric acid; C14: myristic acid; C14:1: myristoleic acid; C15 iso: isopentadecylic acid; C15 anteiso: antiesopentacyclic acid; C16:1w7: palmitoleic acid; C16:1w5: n-hexadecanoic acid; C16: palmitic acid; C17: margaric acid; C17 iso: isomargaric acid; C17 anteiso: anteisomargaric acid; C17:1: heptadecenoic acid; C18: stearic acid; C18:1w9: oleic acid; C18:1w7: vaccenic acid; C18:2w6: linoleic acid; C18:3w6: gamma-linolenic acid; C20: arachidic acid; C20:1w9: eicosenoic acid.

**Table 3:** Effects of gamma irradiation on the fatty acid composition of *S. sonnei*.

	Fatty Acids		
Strains	SFA	UFA	UFA/SFA
Strain non-irradiated	43.08 ± 0.255	60.09 ± 0.030	1.39 ± 0.007
Strain irradiated at 0.5 kGy	37.38 * ± 0.105	60.21 * ± 0.026	1.61 * ± 0.013
Strain irradiated at 1 kGy	36.72 * ± 0.025	65.38 * ± 0.022	1.78 * ± 0.011

*: *p* < 0.05; SFA: Total saturated fatty acids; UFA: Total unsaturated fatty acids.

**Table 4:** Energy of interaction estimated by a docking study of FAs in the VirF binding cavity.

Common Name	Structure	Carbon Atoms: Double Bonds	Energy
Capric acid	CH_3_(CH_2_)_8_COOH	10:0	−5.0
Lauric acid	CH_3_(CH_2_)_10_COOH	12:0	−5.4
Myristic acid	CH_3_(CH_2_)_12_COOH	14:0	−5.6
13-methyltetradecanoic acid	(CH_3_)_2_CH(CH_2_)_11_COOH	15:0 iso	−5.6
12-methyltetradecanoic acid	(CH_3_)_2_CH(CH_2_)_11_COOH	15:0 antieso	−5.9
Palmitic acid	CH_3_(CH_2_)_14_COOH	16:0	−5.7
Margaric acid	CH_3_(CH_2_)_15_COOH	17:0	−5.7
15-methylehexadecanoic acid	(CH_3_)_2_CH(CH_2_)_13_COOH	17:0 iso	−5.9
14-methylehexadecanoic acid	(CH_3_)_2_CH(CH_2_)_13_COOH	17:0 antieso	−6.0
Stearic acid	CH_3_(CH_2_)_16_COOH	18:0	−5.8
Arachidic acid	CH_3_(CH_2_)_18_COOH	20:0	−5.7
Myristoleic acid	CH_3_(CH_2_)_3_CH=CH(CH_2_)_7_COOH	14:1	−5.4
Palmitoleic acid	CH_3_(CH_2_)_5_CH=CH(CH_2_)_7_COOH	16:1w7	−5.9
Hexadecanoic acid	CH_3_(CH_2_)_3_CH=CH(CH_2_)_9_COOH	16:1w5	−5.8
Margaroleic acid	CH_3_(CH_2_)_6_CH=CH(CH_2_)_7_COOH	17:1	−5.9
Oleic acid	CH_3_(CH_2_)_7_CH=CH(CH_2_)_7_COOH	18:1w9	−6.3
Gondoic acid	CH_3_(CH_2_)_7_CH=CH(CH_2_)_9_COOH	20:1w9	−6.2
Linoleic acid	CH_3_(CH_2_)_4_CH=CH(CH_2_)CH=CH(CH_2_)_7_COOH	18:2w6	−6.5
Gamma-linolenic acid	CH_3_(CH_2_)_4_CH=CH(CH_2_)CH=CH(CH_2_)CH=CH(CH_2_)_4_COOH	18:3w6	−6.5

## Data Availability

The original contributions presented in this study are included in the article. Further inquiries can be directed to the corresponding authors.

## References

[1] Gaougaou G., Shankar S., Liot Q., Constant P., Déziel E., Lacroix M. (2020). Gamma irradiation triggers a global stress response in *Escherichia coli* O157:H7 including base and nucleotides excision repair pathways. Microb. Pathog..

[2] Khan J., Bhattacharya S.K. (2017). Shigellosis in a Newborn—An Uncommon Case. Int. J. Biomed. Eng. Clin. Sci..

[3] Miles S.L., Holt K.E., Mostowy S. (2024). Recent advances in modelling *Shigella* infection. Trends Microbiol..

[4] Kaniga K., Trollinger D., Galan J.E. (1995). Identification of two targets of the type III protein secretion system encoded by the inv and spa loci of *Salmonella typhimurium* that have homology to the Shigella IpaD and IpaA proteins. J. Bacteriol..

[5] Nyarkoh R., Odoom A., Donkor E.S. (2024). Prevalence of *Shigella* species and antimicrobial resistance patterns in Africa: Systematic review and meta-analysis. BMC Infect. Dis..

[6] Warren B.R., Parish M.E., Schneider K.R. (2006). *Shigella* as a Foodborne Pathogen and Current Methods for Detection in Food. Crit. Rev. Food Sci. Nutr..

[7] Chitose N., Ueta S., Seino S., Yamamoto T.A. (2003). Radiolysis of aqueous phenol solutions with nanoparticles.1. Phenol degradation and TOC removal in solutions containing TiO_2_ induced by UV, γ-ray and electron beams. Chemosphere.

[8] Wei Y., Van Houten R.T., Borger A.R., Eikelboom D.H., Fan Y. (2003). Minimization of excess sludge production for biological wastewater treatment. Water Res..

[9] Bougrier C., Albasi C., Delgenès J.P., Carrère H. (2006). Effect of ultrasonic, thermal and ozone pre-treatments on waste activated sludge solubilisation and anaerobic biodegradability. Chem. Eng. Process. Process Intensif..

[10] Dutta D., Arya S., Kumar S. (2021). Industrial wastewater treatment: Current trends, bottlenecks, and best practices. Chemosphere.

[11] Borrely S.I., Cruz A.C., Del Mastro N.L., Sampa M.H.O., Somessari E.S. (1998). Radiation processing of sewage and sludge. A review. Prog. Nucl. Energy.

[12] Wang J., Wang J. (2007). Application of radiation technology to sewage sludge processing: A review. J. Hazard. Mater..

[13] Guillén S., Nadal L., Álvarez I., Mañas P., Cebrián G. (2021). Impact of the Resistance Responses to Stress Conditions Encountered in Food and Food Processing Environments on the Virulence and Growth Fitness of Non-Typhoidal *Salmonellae*. Foods.

[14] Lee N.Y., Jo C., Shina D.H., Kim W.G., Byun M.W. (2006). Effect of g-irradiation on pathogens inoculated into ready-to use vegetables. Food Microbiol..

[15] Gomes T., Song Y., Brede D.A., Xie L., Gutzkow K.B., Salbu B., Tollefsen K.E. (2018). Gamma radiation induces dose-dependent oxidative stress and transcriptional alterations in the freshwater crustacean *Daphnia magna*. Sci. Total Environ..

[16] Jann K., Schmidt G., Blumanstock E., Vosbeck K. (1981). *Escherichia coli* adhesion to *Saccharomyces cerevisiae* and mammalian cells: Role of piliation and surface hydrophobicity. Infect. Immun..

[17] Palomar J., Leranoz A.M., Vinas M. (1995). *Serratia marcescens* adherence: The effect of O antigen pressure. Microbios.

[18] Ellafi A., Denden I., Abdallah F.B., Souissi I., Bakhrouf A. (2009). Survival and adhesion ability of *Shigella* spp. strains after their incubation in seawater microcosms. World J. Microbiol. Biotechnol..

[19] Vu D.T., Sethabutr O., Von Seidlein L., Tran V.T., Do G.C., Bui T.C., Le H.T., Lee H., Houng H.S., Hale T.L. (2004). Detection of Shigella by a PCR assay targeting the ipaH gene suggests increased prevalence of shigellosis in Nha Trang, Vietnam. J. Clin. Microbiol..

[20] Hartman A.B., Venkatesan M.M., Oaks E.V., Buysse J.M. (1990). Sequence and molecular characterization of multicopy invasion plasmid antigen gene, ipaH, of *Shigella flexneri*. J. Bacteriol..

[21] Trott O., Olson A.J. (2010). AutoDock Vina: Improving the speed and accuracy of docking with a new scoring function, efficient optimization, and multithreading. J. Comput. Chem..

[22] Midgett C.R., Talbot K.M., Day J.L., Munson G.P., Kull F.J. (2021). Structure of the master regulator Rns reveals an inhibitor of enterotoxigenic *Escherichia coli* virulence regulons. Sci. Rep..

[23] Maurelli A.T., Sansonetti P.J. (1988). Identification of a chromosomal gene controlling temperature-regulated expression of *Shigella* virulence. Proc. Natl. Acad. Sci. USA.

[24] Villavicencio A.L.C.H., Araújo M.M., Marin-Huachaca N.S., Mancini-Filho J., Delincée H. (2004). Identification of irradiated refrigerated poultrywith the DNA comet assay. Radiat. Phys. Chem..

[25] Lee J.L., Levin R.E. (2008). New approach for discrimination of *Vibrio vulnificus* by real-time PCR before and after γ-irradiation. J. Microbiol. Methods.

[26] Teixeira H., Gonçalves M.G., Rozès N., Ramos A., San Romão M.V. (2002). Lactobacillic Acid Accumulation in the Plasma Membrane of *Oenococcus oeni*: A Response to Ethanol Stress?. Microb. Ecol..

[27] Caillet S., Millette M., Dussault D., Shareck F., Lacroix M. (2008). Effect of gamma radiation on heat shock protein expression of four foodborne pathogens. J. Appl. Microbiol..

[28] Lim S., Jung J., Kim D. (2007). The effect of gamma radiation on the expression of the virulence genes of *Salmonella typhimurium* and *Vibrio* spp.. Radiat. Phys. Chem..

[29] Russell N.J., Fukunaga N. (1990). A comparison of thermal adaptation of membrane lipids in psychrophilic and thermophilic bacteria. FEMS Microbiol. Lett..

[30] Rotger R., Casadesu’s J. (1999). The virulence plasmids of *Salmonella*. Int. Microbiol..

[31] Touchon M., Rocha E.P. (2007). Causes of insertion sequences abundance in prokaryotic genomes. Mol. Biol. Evol..

[32] Ben Abdallah F., Bakhrouf A., Ayed A., Kallel H. (2009). Alterations of outer membrane proteins and virulence genes expression in gamma-irradiated *Vibrio parahaemolyticus* and *Vibrio alginolyticus*. Foodborne Pathog. Dis..

[33] McVicker G., Tang C.M. (2016). Deletion of toxin-antitoxin systems in the evolution of Shigella sonnei as a host-adapted pathogen. Nat. Microbiol..

[34] Trirocco R., Pasqua M., Tramonti A., Grossi M., Colonna B., Paiardini A., Prosseda G. (2023). Fatty Acids Abolish *Shigella* Virulence by Inhibiting Its Master Regulator, VirF. Microbiol. Spectr..

[35] Ogawa M., Yoshimori T., Suzuki T., Sagara H., Mizushima N., Sasakawa C. (2005). Escape of intracellular *Shigella* from autophagy. Science.

[36] Kotloff K.L., Riddle M.S., Platts-Mills J.A., Pavlinac P., Zaidi A.K.M. (2018). Shigellosis. Lancet.

[37] Baker S. (2018). The HC Recent insights into. Curr. Opin. Infect. Dis..

[38] Cortés-Avalos D., Martínez-Pérez N., Ortiz-Moncada M.A., Juárez-González A., Baños-Vargas A.A., Estrada-de Los Santos P., Pérez-Rueda E., Ibarra J.A. (2021). An update of the unceasingly growing and diverse AraC/XylS family of transcriptional activators. FEMS Microbiol. Rev..

[39] Bodero M.D., Harden E.A., Munson G.P. (2008). Transcriptional regulation of subclass 5b fimbriae. BMC Microbiol..

[40] Di Martino M.L., Falconi M., Micheli G., Colonna B., Prosseda G. (2016). The Multifaceted Activity of the VirF Regulatory Protein in the *Shigella* Lifestyle. Front. Mol. Biosci..

[41] Mitchell M.K., Ellermann M. (2022). Long Chain Fatty Acids and Virulence Repression in Intestinal Bacterial Pathogens. Front. Cell. Infect. Microbiol..

[42] Plecha S.C., Withey J.H. (2015). Mechanism for inhibition of *Vibrio cholerae* ToxT activity by the unsaturated fatty acid components of bile. J. Bacteriol..

